# Bioinformatic Tools for Studying the Cellular Immune Response to SARS-CoV-2, Vaccine Efficacy, and Future Pandemics at the Global Population Level

**DOI:** 10.3390/ijms252413477

**Published:** 2024-12-16

**Authors:** Daniel López, Javier Zumárraga

**Affiliations:** Centro Nacional de Microbiología, Instituto de Salud Carlos III, 28220 Majadahonda, Madrid, Spain; javizfranco@gmail.com

**Keywords:** cellular response, HLA, immunoinformatics, SARS-CoV, vaccine

## Abstract

Antigen recognition by human leukocyte antigen (HLA) restriction is critical for an adequate antiviral response in both natural infection and vaccination. However, the overwhelming polymorphism of HLA, with nearly 40,000 alleles identified, is an important limitation for the global analysis of cellular immune responses and vaccine efficacy. In this narrative review, we included several immunoinformatics studies performed in our laboratory to circumvent this limitation. These analyses focused on studying the cellular immune responses restricted by the most common HLA alleles, and their role in vaccine efficacy. Computational studies validated experimentally, such as our laboratory has carried out, represent a useful, rapid, and cost-effective strategy to combat future pandemics.

## 1. Introduction

The three arms of adaptive immunity (B lymphocytes associated with the humoral immune response, helper CD4^+^ T cells, and cytolytic CD8^+^ T lymphocytes linked to the cellular immune response) are essential for surviving natural virus infection and generating a protective immune response following vaccination [1]. Furthermore, in mouse models, depletion of CD4^+^ and CD8^+^ T lymphocytes results in persistent replication without viral clearance in the nasal compartment and an increase in adaptive mutations throughout the SARS-CoV-2 genome [2]. In germinal centers, naïve B cells interact with the virus antigen in its native form and follicular CD4^+^ T helper lymphocytes, triggering proliferation, class-switch recombination, and affinity maturation of B lymphocytes, which are ultimately differentiated into mature B cells that secrete antibodies with high affinity. In contrast, precise recognition of denatured short viral peptides bound to human leukocyte antigen (HLA) class I or II molecules by CD8^+^ or CD4^+^ T cell receptors (TCRs) is essential in the cellular immune response. Single changes in the viral peptide sequence determine functional or non-functional interaction with the TCRs, which, if productive, triggers multiple activities of these lymphocytes and other cells of the immune system. For example, these functions include conversion of resting T cells in mature effector lymphocytes, stimulation of B cells’ growth and their differentiation to plasma cells, secretion of cytokines to attract different subpopulations of the immune system as macrophages, neutrophils, and other T cells, and the generation of immunological memory, among many others [3]. In addition to the many strategies for immune evasion used by viruses (down-regulation of HLA expression, release of immunomodulatory substances, etc.), the lack of efficient antigen recognition by HLA-restricted class I and class II T cells means that the immune system is not properly activated, causing the virus to spread throughout the body [4], with potentially fatal consequences for the host [5].

## 2. HLA Class I and II Restriction for Antigen Processing and Presentation

**Figure 1:**
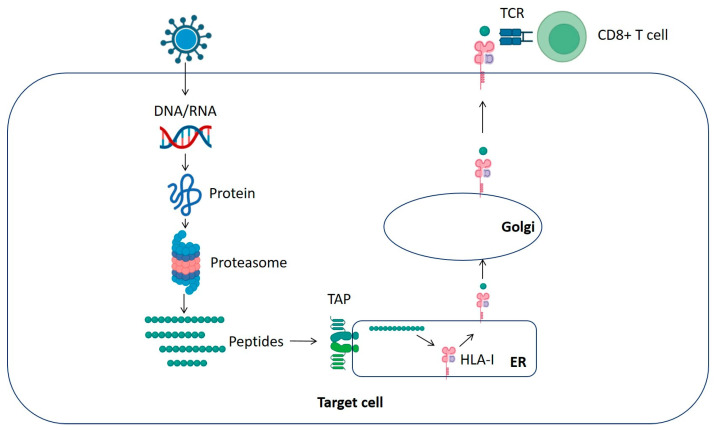
HLA class I antigen processing and presentation pathway. Diagram demonstrating the production of peptides for HLA class I presentation. Endogenous pathway: viral peptides processed by proteasomes and translocated to the ER by transporter associated with antigen processing (TAP) bind to HLA class I molecules and are transported to the cell surface, where they interact with CD8^+^ T cells.

In infected cells, viral proteins with synthesis or folding defects are targeted for proteolytic degradation by cytosolic proteases, mainly different types of proteasomes (standard, single intermediate proteasome, double intermediate proteasome, immunoproteasome, thymoproteasome, and spermatoproteasome, differing in subunit composition and cleavage specificity), generating a wide range of peptides of different sequences and lengths. Subsequently, some of these processed peptides are specifically translocated into the lumen of the endoplasmic reticulum (ER) by transporters associated with antigen processing. Peptides of 8 to 12 residues in length are efficiently translocated, but this transport becomes increasingly detrimental as the peptide length increases. Those peptides with the correct length (8 to 11 residues) and productive molecular interactions with specific residues of the antigen-binding site of the HLA class I molecule [6] can bind and stabilize the newly synthesized HLA class I molecules. These class I peptide/HLA complexes are exported through the endomembrane system to the cell surface, where they can interact with CD8^+^ T-cell receptors (Figure 1) [7]. Efficient recognition of viral ligands bound to HLA class I molecules by these CD8^+^ T lymphocytes can lead to the beneficial death of pathogen-infected cells as usual in protective antiviral immune responses or, conversely, induce autoimmune damage such as HLA-B27-restricted autoreactive cytolytic T cells associated with ankylosing spondylitis, psoriatic arthritis, and other spondyloarthropathies [8].

**Figure 2:**
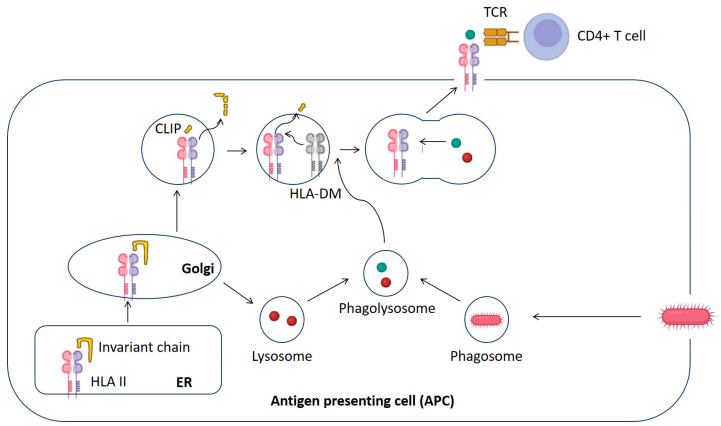
HLA class II antigen processing and presentation pathway. Diagram demonstrating the production of peptides for HLA class II presentation. Exogenous pathway: HLA class II molecules associated with the invariant chain in the ER are transported to endosomal compartments where the invariant chain is degraded and exogenous peptides bind, allowing interaction with CD4^+^ T cells on the cell surface.

In contrast, HLA class II molecules synthesized by antigen-presenting cells associate with the invariant (Ii) class II chain in the endoplasmic reticulum. These stabilized complexes, without bound antigenic peptides, are transported directly to different specialized endosomal compartments where the Ii chain is progressively proteolyzed until only a fragment known as the class II-associated invariant chain peptide (CLIP) remains. CLIP interacts with the antigen-binding site of the HLA class II molecule to prevent binding of pathogenic peptides from endogenous HLA class I pathways. Next, interaction of CLIP/HLA class II complexes with the accessory molecule HLA-DM induces conformational changes in the HLA class II molecules, and subsequent release of CLIP from this HLA molecule exposes the antigenic peptide binding site. This HLA-DM protein acts as a chaperone that stabilizes HLA class II molecules without CLIP, to ensure binding to high-affinity peptides. This organelle fuses with late endosomes containing exogenous protein material, such as extracellular host proteins and/or viral particles previously engulfed by phagocytosis, endocytosis, or pinocytosis. This exogenous material is processed by various lysosomal resident cathepsins. This results in viral peptides of varying length [9]. Finally, the binding of these antigenic peptides of different lengths (up to 30 residues long), which interact productively with specific residues of the antigen-binding site of the HLA class II molecule, stabilizes these class II peptide/HLA complexes, allowing their subsequent transport to the cell membrane, where they can be recognized by CD4^+^ T lymphocyte receptors (Figure 2) [9]. The recognition of viral ligands bound to HLA class I molecules triggers multiple immune activities, including secretion of cytokines, B-cell antibody class switch, and the growth and activation of cytolytic T lymphocytes, thereby increasing the bactericidal activity of phagocytic cells.

In summary, HLA class I antigen processing serves to monitor the entire proteome of any cell in the organism, whereas its class II counterpart is associated with proteins of extracellular origin processed by antigen-presenting cells. In addition, HLA class II-restricted antigen presentation has the primary effector function of eliminating pathogen-infected cells, whereas HLA class II serves primarily to initiate and regulate the entire subsequent adaptive immune response.

## 3. Computational Tools Can Address HLA Polymorphism, a Major Impediment to the Study of Cellular Immune Response and Vaccine Efficacy at the Global Human Population Level

**Figure 3:**
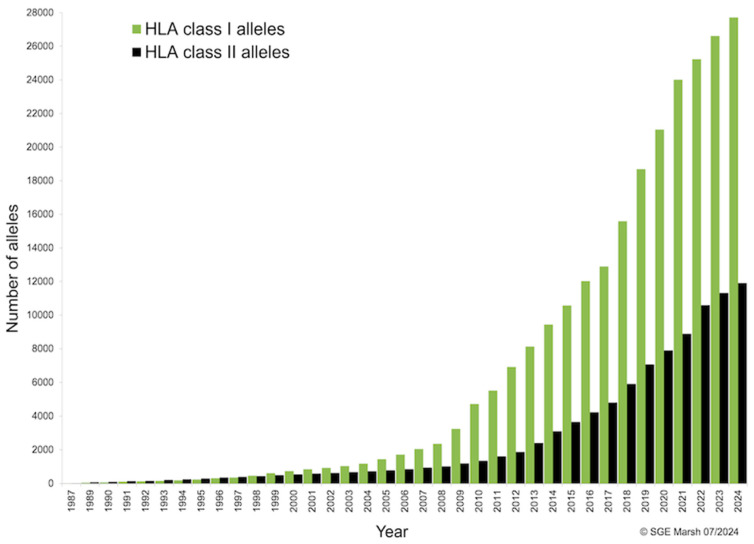
Identification of HLA class I and II genetic polymorphisms by year. Number of alleles named and included in the IPD-IMGT/HLA Database since 1987 [13].

Since the first tens of HLA alleles were identified in the late 1980s, the number of HLA class I and II variants sequenced has followed an almost exponential growth curve with no plateau in sight. This may simply be because current sequencing capacity is far superior to that of a few decades ago, but the fact that new HLA alleles continue to be identified even in well-studied populations such as Caucasians in Europe [10,11,12] suggests that there are still many HLA polymorphisms to be discovered. Thus, with nearly 40,000 alleles currently identified (27,300 for class I and 11,600 for HLA class II), the HLA genes show by far the highest degree of polymorphism in the human genome (Figure 3).

Since most of the amino acid changes that generate this amazing array of different proteins are located in specific residues of the antigen-binding sites of these tens of thousands of HLA molecules, each HLA protein variant in principle has unique peptide-binding properties, which can have a significant impact on the control of some viral infections. For example, two HLA-B*35 subtypes that differ by a single amino acid change differentially bind several HIV peptides, leading to very different rates of progression to AIDS [14]. Therefore, experimental analysis of the cellular immune response and its relevance to vaccine efficacy at the global population level is extremely difficult. However, HLA alleles with limited sequence changes can share strong peptide–ligand specificities and bind to highly overlapping peptide sets (i.e., HLA ligandomes). Based on similarities in their structure and functionality that have been identified in experiments conducted over decades by several hundred research groups using different methodological approaches (e.g., sequencing and crystallography of HLA alleles, and mass spectrometry identification of tens of thousands of HLA ligands), many related antigen-presenting alleles have been progressively grouped at three different levels: families, superfamilies, and canonical HLA class I or II supertypes. This superfamily classification covers >90% of the world’s population, regardless of ethnicity [15,16,17]. This enables bioinformatics approaches that analyze cellular immune responses and their relationship with vaccination at the global population level; such studies cannot feasibly be conducted through traditional experimental methods. In parallel, open resources such as IEDB have collected hundreds of thousands of HLA class I and II epitopes published in tens of thousands of scientific papers [18]. This wealth of information has enabled the development of algorithms and machine learning models that can effectively predict the processing, binding, and immunogenicity of T-cell epitopes associated with essentially all of the documented HLA alleles most frequently found in the human population. NetMHCpan and NetMHCIIpan are the most efficient methods to assess binding to HLA class I and II molecules, respectively; both are recommended and utilized by the IEDB. They consist of artificial neural networks trained with experimental peptide sequences from either eluted epitopic peptides identified by mass spectrometry or known allele-dependent binding affinities. More than 17 million data points covering 370 different HLA class I and II molecules can be used for training. Once trained, these algorithms can predict the binding of experimentally undetected protein peptides and assign binding affinities as formally measured biochemically (i.e., calculating the half maximal inhibitory concentration, IC50).

In recent years, our laboratory has used these types of bioinformatics tools to analyze various aspects of the cellular immune response to SARS-CoV-2 and the potential cellular efficacy of vaccines against this pathogen at the global population level.

## 4. Assessment of the Effect of Viral Mutations on the Cytotoxic Response to SARS-CoV-2 in the Global Human Population

Since its discovery, laboratories around the world have reported the enormous capacity for variation of the pathogen that caused the COVID-19 pandemic. In general, these mutations can have different effects: altering/enhancing the infectious capacity or transmissibility of SARS-CoV-2 [19] or interfering with/reducing the immune response by either antibodies [20] or evading T-cell killing by disrupting peptide–MHC binding or TCR recognition [21] against this pathogen. As with many other viral pathogens, an adequate cytolytic T-cell response has been proven effective in controlling both primary and subsequent SARS-CoV-2 infections through the generation of long-term protection [1,2,22].

Many bioinformatics analyses have been performed to predict T-cell-recognized epitopes associated with some of the most common HLA alleles in the population (reviewed in [23]). Although some of those analyses were similar to those performed by our group when investigating T-cell responses to SARS-CoV-2, they included analysis of far fewer HLA alleles [24,25]. Additionally, a comprehensive relationship between the striking HLA polymorphisms and the immense genetic variability of SARS-CoV-2 remained unaddressed.

Bearing this in mind, we bioinformatically predicted the HLA-restricted epitopes of the SARS-CoV-2 proteome presented by more than 2900 human alleles belonging to 71 different HLA class I families [26]. In this study, we also considered all amino acid changes identified in approximately 118,000 NCBI viral isolates sequenced to that date, identified in more than 80 countries, covering virtually all subcontinental areas of the United Nations M49 Geosystem. This large matrix of HLA alleles and SARS-CoV-2 variants revealed mutations that could affect HLA class I binding at the global population level, identifying potential escape mutations of the cellular cytolytic response worldwide, as well as possible low vaccine efficacy or even vaccine failure [26].

Compared with the original Wuhan-1 reference strain, more than one million mutations that had altered the amino acid sequence of coronaviral proteins were identified in that study [26]. More than 75% of these identified residue changes were non-conservative. Those mutations that significantly reduced HLA binding affinity and had a wide geographic distribution of isolates were considered potential escape mutations capable of decreasing the cytotoxic immune response globally and warranting surveillance. We found that 22% of the epitopes analyzed contained these worrying mutations and that the areas “Australia and New Zealand” and especially “North America” had multiple viral isolates with the highest rates of escape mutations [26].

In addition, this study predicted that 8% (246 alleles) of the analyzed HLA class I molecules could present few high-affinity epitopes from the SARS-CoV-2 proteome to cytotoxic T lymphocytes. Some of these alleles are widely distributed throughout Africa and Eastern Asia. Likewise, immigrant populations from these places are also relevant in Western countries such as Germany and the USA. As these inefficient alleles are often found in the same HLA haplotype as others capable of binding many more high-affinity epitopes, the overall cytotoxic immune response of these individuals should not be affected. However, individuals with the A*74:02-B*46:01-C*01:02 haplotype express some of these “poor” SARS-CoV-2 epitope-presenting alleles at all three loci [26]. This haplotype has been detected at low frequency in the densely inhabited country of China and could be the subject of a more detailed study to compare cellular immune responses to COVID-19 and the survival rate of its carriers versus people with other haplotypes. These results show that this type of bioinformatics analysis can be used to formulate very specific hypotheses from global data, which could be of great importance for public health.

## 5. Bioinformatics Analyses of the Influence of Emerging SARS-CoV-2 Variants on the Efficacy of COVID-19 Vaccines at the Global Population Level

In the spring of 2020, the COVID-19 pandemic became a worldwide public health emergency, with hospitals under severe pressure due to the exponential growth of infections, patients, and deaths on all continents. This unprecedently mobilized thousands of scientists, several multinational pharmaceutical corporations, and numerous public and private health institutions around the world to produce vaccines to control this deadly epidemic in the shortest possible time. Beginning in late 2021, the WHO began to authorize the emergency use of at least 12 COVID-19 vaccines (https://covid19.trackvaccines.org/agency/who/, accessed on 1 November 2024). All of these formulations were based on the sequence of the D614 spike protein from the first publicly available SARS-CoV-2 genome data, as envelope glycoproteins have historically proven to be ideal targets for vaccines against a wide variety of viruses.

Although the desirable characteristics for an ideal vaccine have been delineated [27], some of the preparations most widely used worldwide during the COVID-19 pandemic failed to meet expectations since they required the administration of multiple doses, did not generate long-term protective immune responses, and required a complex cold chain. For previously mentioned reasons, administration and distribution of these vaccines was logistically difficult, especially in developing countries, and this should be considered by regulatory and public health agencies should a similar circumstance arise.

However, even before the first COVID-19 vaccines were approved for human use, an increasing number of SARS-CoV-2 variants and subvariants were identified in different parts of the world [19]. Thus, health authorities in numerous countries, as well as the WHO, expressed concern about the efficacy of vaccines (under development or recently licensed) against new strains already identified and specifically, future variants and subvariants of SARS-CoV-2, and their impact on the control of COVID-19.

As noted above, the extraordinary polymorphism of HLA makes it extremely difficult to analyze the cellular immune response and its relationship to vaccine efficacy at the global population level. By focusing on the HLA-restricted immune response, we addressed this problem bioinformatically [28]. First, we included all SARS-CoV-2 variants with at least 100 genomes included in the GISAID emerging variant tracking tool prior to June 2021. Later, we analyzed the possible impact of the SARS-CoV-2 mutations included in these genomes that could facilitate immune escape from the cytolytic immune response of the 551 HLA class I alleles most commonly found in humans and covering > 90% of the world population, independent of ethnicity [15]. The vast majority of these SARS-CoV-2 proteomes had an HLA-restricted escape epitope rate of less than 10%. The exception were some British and South Africa variants with higher escape epitope rates for HLA class I alleles included in the B07 and B27 supertypes, respectively [28]. However, all SARS-CoV-2 variants tested still contained sufficient epitopes without escape mutations for vaccines produced from the Wuhan strain to maintain the vast majority of cytolytic cellular immune protection at the global population level [28].

At the end of November 2021, a new emerging variant of concern, first detected in Botswana, spread rapidly, first across the African continent and then around the world. [29]. This Omicron variant had multiple nonsynonymous genome mutations, accumulating 37 amino acid changes in the spike protein compared with the Wuhan-1 sequence used in all immunogenic formulations, raising questions about the efficacy of COVID-19 vaccines [30].

Therefore, we performed an immunoinformatics analysis of amino acid changes included in the Omicron spike protein that altered the immune response elicited by licensed vaccines in response to this emerging SARS-CoV-2 variant [31]. For this study, we computationally analyzed both the cytolytic and helper immune responses restricted by the 551 and 41 most prevalent HLA class I and II alleles, respectively. These 592 HLA class I and II molecules cover > 90% of the human population across the world [15,16,17] and thus provide a complete picture of vaccine efficacy concerning the human population’s ability to develop a complete cellular response. For the majority of the nearly 600 HLA alleles analyzed, the amino acid differences in the spike proteins of the Omicron variant of concern and the Wuhan-1 strain resulted in very high conservation (90%) of HLA-restricted class I or II epitopes, as an average of 13.5 out of 15 epitopes per allele predicted in Wuhan-1 were conserved in Omicron [31]. We identified only three HLA-B*48 subtypes (HLA-B*48:01, -B*48:04, and -B*48:05) that had only two predicted epitopes in the vaccine sequence and in which Omicron amino acid changes completely eliminated the cytolytic cellular immune response induced by the vaccines [31]. These HLA-B alleles are only relevant in some very minor ethnic groups. Specifically, HLA-B*48:01 is present in approximately 20–25% of the Amis, Atayal, and Taroko indigenous peoples of Taiwan, in 10–22% of various Amerindian populations from Peru and USA, and Maori populations from American Samoa and New Zealand. The frequency of the HLA-B*48:04 and -B*48:05 alleles is much lower still (<1%) in some ethnic subpopulations in China, India, Israel, Jordan, and Saudi Arabia. Together, these three HLA-B*48 alleles are present in less than one million people worldwide, indicating that the negative impact of Omicron on the cytolytic T-cell immune responses associated with COVID-19 vaccines should be close to negligible [31]. These facts are consistent with several experimental studies that have analyzed the cellular immune responses generated by the SAR-CoV-2 vaccines, using cohorts of less than 100 individuals each from the Netherlands [32], Japan [33], the United States [34,35], and South Africa [36].

Furthermore, our findings are also especially relevant in the case of vaccinated individuals with an inherited (X-linked agammaglobulinemia) or acquired (treatment with anti-CD20 antibodies) lack of mature B lymphocytes. Immunocompromised patients in whom protection against SARS-CoV-2 infection depends on HLA-mediated cellular immune responses developed a functional spectrum of SARS-CoV-2-restricted effector and memory CD8^+^ T cells similar to that of the general population, with a modest reduction in follicular helper and memory CD4^+^ T lymphocytes [37,38].

## 6. Is It Possible That Future SARS-CoV-2 Variants Will Evade the T-Cell-Mediated Immune Responses Generated by the Current COVID-19 Vaccines?

Our previous work showed that the cytotoxic and helper immune responses generated by the COVID-19 vaccines would hardly be affected by the identified emerging variants of SARS-CoV-2, at least up to the Omicron strain [28,31]. However, this coronavirus is constantly evolving and accumulating mutations relative to the original Wuhan-1 strain [39]. An obvious question then arises. How far must SARS-CoV-2 evolve before current vaccines are no longer effective for most of the human population in terms of cellular immune response?

**Figure 4:**
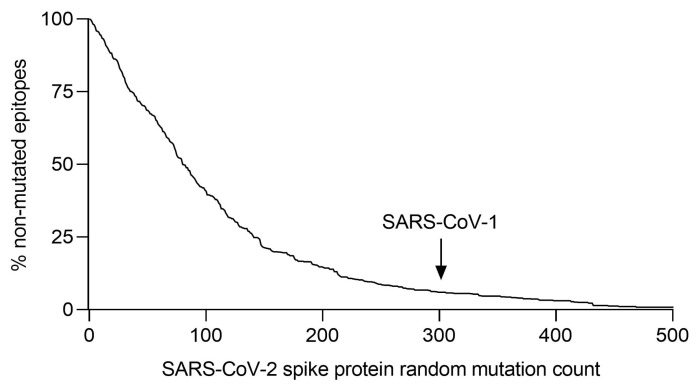
Percentage of conserved epitopes in the SARS-CoV-2 spike protein after increasing random mutagenesis. The number of non-mutated epitopes for the 551 HLA class alleles most prevalent in the human population was calculated after random progressive position sets ranging from 1 and 500 positions, i.e., the length of the full spike protein (adapted from [28]). For comparison, the arrow indicates the 304 amino acid changes between SARS-CoV-1 and SARS-CoV-2.

To attempt to answer this question, we computationally generated three sets of progressive random mutations over the spike protein sequence of the Wuhan-1 strain, and the accumulated loss of HLA epitopes for each new hypothetical mutant was analyzed [28]. This assay was based on the worst-case hypothesis, i.e., that any change in the amino acid in an epitope, even if it does not necessarily abolish binding to the HLA molecule on the target cell, will disrupt the TCR recognition of memory CD8^+^ cells [40]. In addition, this computational analysis took into account that an amino acid change that destroys an epitope for one HLA allele could create a new epitope for another HLA allele. This in silico experiment showed that, on a global human population scale, 34 mutations destroyed 25% of the epitopes generated by the vaccines, 80 amino acid changes removed half of these epitopes, and 140 mutations still left 25% of the original epitopes unchanged (Figure 4). Keeping in mind that short deletions in the protein can also destroy epitopes, it would take 235 amino acid mutations in the Wuhan-1 sequence to eliminate 90% of the HLA epitopes of the COVID-19 vaccines (Figure 4). These mutations are much higher than those currently found in any strain of concern and are probably higher than in any other SARS-CoV-2 strain that may evolve in the future. Therefore, because a given virus cannot accumulate mutations ad infinitum (as indicated the 304 changes between SARS-CoV-1 and SARS-CoV-2, Figure 4), the original COVID-19 vaccines would still be able to generate a significant T-cell immune response against any future variant of concern. In addition, unlike in our bioinformatics experiment, mutations of SARS-CoV-2 (like those of all viruses) are not evenly distributed, as many of these real mutations are concentrated in specific protein regions; thus, fewer epitopes are affected. Therefore, large regions of the spike protein remain unchanged, which means that our results are a very conservative estimate of the potential loss of HLA epitopes in future SARS-CoV-2 mutant strains.

## 7. COVID-19 Vaccines Generate T-Cell Cross-Protection Against SARS-CoV-1

**Figure 5:**
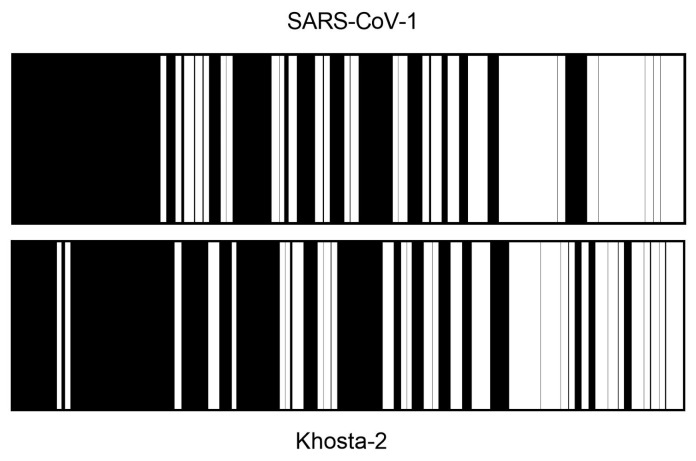
Conservation of SARS-CoV-2 spike protein with its SARS-CoV-1 and Khosta-2 homologs. Conserved (white) or non-conserved (black) segments of ≥9 consecutive residues in the SARS-CoV-2 spike protein and its homologous proteins in SARS-CoV-1 (**upper** panel) or Khosta-2 (**bottom** panel) viruses (adapted from [42]).

In our previous computational prediction using progressive random mutations [28], it was found that 304 amino acid changes in the SARS-CoV-2 spike protein (same as the SARS-CoV-1 glycoprotein) would share only 6% of the conserved epitopes with the SARS-CoV-2 vaccine strain (Figure 4). As a result, there would be no theoretical possibility of T-cell cross-protection between the COVID-19 vaccines and subsequent infection with SARS-CoV-1. However, as noted above, the amino acid changes between the spike proteins of both sarbecoviruses were not randomly distributed across these protein sequences. Therefore, in a real scenario, we further analyzed the possible cytotoxicity and/or helper cross-recognition mediated by HLA class I and II restricted epitopes conserved between the sequenced SARS-CoV-1 and SARS-CoV-2 spike proteins [41]. Bioinformatic prediction of the 608 HLA class I and II alleles, which are found in >90% of the human population, revealed that 31% of the predicted epitopes in the COVID-19 vaccines retained a completely identical sequence in the SARS-CoV-1 spike protein [41]. Thus, as indicated above for the case of future SARS-CoV-2 mutant strains, in the progressive random mutation analysis, we made a very low estimate of potentially conserved T-cell epitopes between the two SARS-CoV viruses. The data indicated that this cautious in silico approach could be improved by introducing parameters associated with virus evolution. For example, the highly exposed regions of viral envelope glycoproteins (e.g., the S1 region of the SARS-CoV-2 spike protein), against which the humoral immune antibody response is predominantly directed, quickly accumulate mutations, in contrast to other regions that are much less immunogenic. Due to this selective pressure of the immune system, there were 27 protein segments of varying length (the longest up to 111 consecutive residues) with complete amino acid identity among the sarbecovirus envelope proteins (Figure 5, upper panel). These protein fragments made up a total of 579 residues where HLA epitopes generated by vaccines against COVID-19 could reactivate memory T cells in the event of subsequent SARS-CoV-1 infection.

The HLA genes are organized within the genome in a superlocus that contains a set of HLA class I (HLA-A, -B, and -C) and class II (HLA-DR, -DP, and -DQ) DNA sequences. This HLA haplotype is a Mendelian inheritance from each parent. Therefore, we bioinformatically analyzed the number of conserved epitopes among sarbecovirus spike proteins for each individual HLA class I and II loci. This computational analysis revealed averages of five, three and seven conserved T-cell epitopes for the HLA-A, -B, and -C loci, respectively. An additional four conserved epitopes for each of the HLA-DR, -DP, and -DQ loci were also identified. Thus, approximately 27 conserved epitopes (15 bound to HLA class I molecules and another 12 associated with HLA class II) could be recognized by T cells in a homozygous individual [41]. In addition, given the fact that more than 85% of the human population is heterozygous for the HLA complex [43], COVID-19 vaccines would be able to generate in these individuals an average of 54 HLA class I and II epitopes against conserved fragments of the SARS CoV-1 spike protein. Furthermore, considering that B cells, antibodies, and regulatory T cells can also cooperate with helper and cytotoxic T cells in a cross-protective immune response, these data strongly suggest that the current vaccines used against COVID-19 should also be effective against a future resurgence of SARS-CoV-1.

## 8. SARS-CoV-1 Vaccines Would Have Provided T-Cell Cross-Protection Against SARS-CoV-2 and Contributed to the Fight Against the COVID-19 Pandemic

Following the first identification of SARS-CoV-2 in the Chinese city of Wuhan, the virus spread rapidly across all continents, and when the number of cases exceeded 100,000 worldwide and there had been 4000 deaths by early 2020, the World Health Organization (WHO) declared the outbreak a public health emergency of international concern (https://www.who.int/director-general/speeches/detail/who-director-general-s-opening-remarks-at-the-media-briefing-on-covid-19---11-march-2020, accessed on 1 November 2024). From that moment on, countless researchers from all over the world, the pharmaceutical industry, and other public and private entities from several countries devoted huge efforts and a large financial investment into developing vaccines to control the pandemic as soon as possible. In the first half of 2021, the WHO and health authorities in several countries began to authorize the emergency use of various COVID-19 vaccines (https://covid19.trackvaccines.org/agency/who/, accessed on 1 November 2024). This drastically reduced the number of infections and deaths caused by COVID-19 [44,45,46]. However, even though these vaccines were developed in record time, could another vaccine have been used to combat COVID-19 even faster and satisfying safety rules better?

In November 2002, SARS-CoV (later renamed SARS-CoV-1) was first identified in Guangdong, China’s most populous province. In the months that followed, infected patients carried the virus first to Hong Kong, Vietnam, and Canada, and then to some 30 countries, with more than 8000 confirmed cases and nearly 800 deaths before it disappeared in May 2004 (https://web.archive.org/web/20200212205529/https://www.who.int/csr/don/2004_05_18a/en/, accessed on 1 November 2024). After 2002 and in subsequent years, several vaccines using inactivated virus or DNA constructs were developed against this pathogen. In various preclinical studies, these vaccines generated high titers of neutralizing antibodies and strong T-lymphocyte immune responses, as well as protective immunity [47,48,49]. Subsequently, Phase I clinical trials of these development-stage vaccines published in 2007–2008 showed no significant side effects in volunteers, and generated robust cellular and humoral immune responses [50,51,52]. In the absence of new outbreaks of this coronavirus, further development of Phase II clinical trials was discontinued.

SARS-CoV-1 and SARS-CoV-2 are two coronaviruses with very high similarity, belonging to the subgenus Sarbecovirus. Our previous bioinformatics study [41] showed that COVID-19 vaccines should be effective against a future resurgence of SARS-CoV-1. Thus, it was obvious that the reverse situation (i.e., that vaccines previously developed against SARS-CoV-1 could cross-react with SARS-CoV-2) should also be true. Thus, we suggest that Phase II clinical trials could have been conducted by early 2020 to determine whether SARS-CoV-1 vaccines could provide partial protection against COVID-19 [53,54]. This very likely scenario would have meant that a vaccine with partial protection would have been available by mid-to-late 2020, most likely reducing COVID-19-associated hospitalization and mortality until the specific SARS-CoV-2 formulations were licensed a year later.

In addition, the history of vaccines had previously demonstrated heterologous protection with different viruses, supporting our hypothesis. First, during the 18th century, various physicians and even a few clever farmers in rural cattle-raising areas of England, Germany, and Holland invented an effective and safe preventive treatment against smallpox, based on the knowledge that milkmaids were not susceptible to this pandemic [55]. Later, Edward Jenner demonstrated the person-to-person protection of this medical procedure [56] and without any knowledge of epidemiology, immunology, or virology, the era of prophylactic vaccines had begun. More than a century later, two poxviruses (cowpox and horsepox) closely related to smallpox were shown to have been used indistinctly in this first empirical vaccination [55]. In addition, the “Smallpox Eradication Program” launched by the WHO in 1959 unintentionally used vaccinia, another virus that is not pathogenic to humans and that also belongs to the same genus of poxviruses as cowpox, horsepox, and smallpox [57]. In addition, some data indicate that even recently detected monkeypox infections can be controlled by prophylactic administration of vaccinia virus [58,59]. Second, mice vaccinated with a recombinant vesicular stomatitis virus (rVSV) expressing the envelope protein of *Reston* or *Taï Forest* ebolaviruses produced complete heterologous cross-protection against challenge with *Zaire ebolavirus* [60]. In addition, in other study, most guinea pigs immunized with an rVSV expressing the *Zaire ebolavirus* glycoprotein generated a cross-protective immune response after challenge with *Sudan ebolavirus* [61]. Thus, the above studies with viruses of the Ebolavirus and Poxvirus genera demonstrate that immunization with closely related viruses can induce significant heterologous immunity.

Finally, Dangi et al. demonstrated in a mouse model that SARS-CoV-1 vaccines generated helper and cytolytic T-lymphocytes that specifically recognized conserved epitopes in the SARS-CoV-2 spike protein, further generating heterologous immunity to challenge [62], as we had hypothesized for the entire human population. In addition, cross-reacting T cells with affinity for SARS-CoV-2 and other human coronaviruses were identified early in the pandemic in individuals not exposed to SARS-CoV-2 [63].

## 9. SARS-CoV-2 Vaccines May Elicit T-Cell Cross-Protection Against the Novel, Potentially Zoonotic Coronavirus Khosta-2

Khosta-2 is a new sarbecovirus identified in two species of horseshoe bats (*R. ferrumequinum* and *R. hipposideros*) living in Sochi National Park in the southwestern Russian Caucasus [64]. The Khosta-2 spike protein binds productively to the human ACE2 receptor and effectively infects human cells, as does its SARS-CoV-2 counterpart [65]. As a result, this new human-transmissible sarbecovirus has raised the concern of international organizations such as the WHO and the fear that it could become a new pandemic. However, analyses of serum from individuals immunized with COVID-19 vaccines showed that the humoral response generated in these individuals was ineffective against Khosta-2 [66]. In this study, sera from individuals who received two doses of COVID-19 vaccine were ineffective in neutralizing the Khosta-2 spike protein [66]. This is not surprising, given that the neutralizing antibodies elicited by the COVID-19 vaccines are primarily directed against the RBD region and the Khosta-2 protein shares only about 60% sequence identity with SARS-CoV-2. It has been shown that the cellular immune responses of cytotoxic and helper T lymphocytes against COVID-19 remain functional even when the antibody immune response induced by the vaccines disappears [67]. Thus, in our laboratory, we wanted to know whether enough HLA class I and class II epitopes were conserved between SARS-CoV-2 vaccines and the Khosta-2 spike protein to induce a cross-reactive cellular immune response. As in our previous study on SARS-CoV-1 and COVID-19 vaccines [41], we determined the number of fully conserved cytotoxic and helper T-cell epitopes among SARS-CoV-2 and Kostha-2 spike proteins for the 567 and 41 most prevalent HLA class I and II alleles, respectively [42]. These computational predictions showed that the overwhelming majority of these 608 alleles, which covers more than 90% of the the world’s population, would be able to present sufficient HLA-restricted conserved epitopes between SARS-CoV-2 and Khosta-2 spike-in proteins to T lymphocytes [42]. Thus, on average for homozygous individuals, 11 epitopes for HLA class I loci and another eight epitopes for HLA class II loci are fully conserved between the Khosta-2 spike proteins and the currently licensed vaccines against COVID-19 (Figure 4) [42]. Therefore, these data strongly suggest that, in relation to epitope binding, current vaccines used against COVID-19 should also be partially effective against future Khosta-2 virus emergence in the immunocompetent human population, with cross-protection generated by the cellular immune response.

In addition, if Khosta-2 were to become a new global pandemic, as SARS-CoV-2 has proven to be, it is very likely that an increasing number of Khosta-2 variants and subvariants could also emerge. As we have shown for SARS-CoV-2, Khosta-2 mutations would not be evenly distributed throughout the spike protein sequence; rather, these mutations would be concentrated in specific regions of the protein and thus, fewer epitopes would be affected. Therefore, large regions of the Khosta-2 spike protein would remain unchanged, meaning that the vaccines would retain an effective response to a significant number of conserved epitopes in future Khosta-2 mutant strains.

## 10. What Is the Accuracy of Computational Prediction of SARS-CoV-2 Epitopes Recognized by T Lymphocytes?

**Figure 6:**
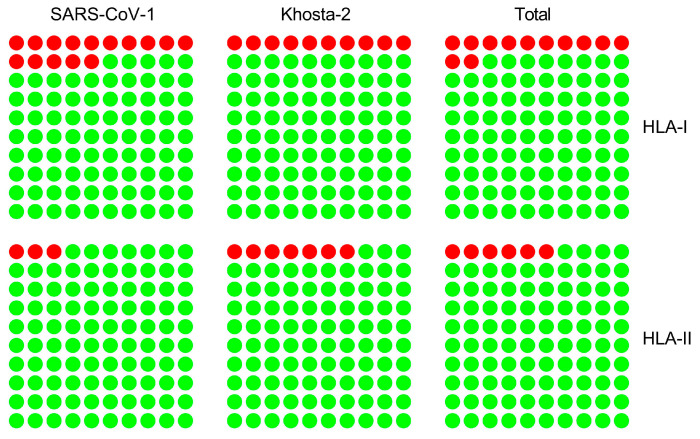
Predicted versus experimentally detected HLA class I and II epitopes conserved among sarbecoviruses. Percentage of predicted experimentally detected HLA class I (**top** panels) or predicted HLA class II (**bottom** panels) epitopes (green dots) or not (red dots) between SARS-CoV-1 and SARS-CoV-2 (**left** panels), Khosta-2 and SARS-CoV-2 (**middle** panels), and the sum of both studies (**right** panels). The HLA class I and II alleles included in this comparison cover > 60% of the world’s population, regardless of ethnic origin. Adapted from [41,42].

Finally, a very important purpose of this type of bioinformatics assay is to determine the performance of the predictive computational identification of real T-cell epitopes in people infected with SARS-CoV-2 or immunized with some of the vaccines currently in use. Since the SARS-CoV-2 pandemic mobilized thousands of laboratories worldwide, the experimental identification of HLA class I and II restricted epitopes from SARS-CoV-2 has grown exponentially, and information is available in various databases such as IEDB [18]. Therefore, we performed a comparison between the experimental data and our immunoinformatic prediction of conserved epitopes between SARS-CoV-1 and SARS-CoV-2 for the most commonly studied HLA class I or II alleles during the coronaviral pandemic. As Figure 6 indicates, the analysis of these 9 HLA class I and 14 HLA class II alleles was congruent with our methodological approach, as we found a very high correlation (85% for HLA class I and 97% for HLA class II epitopes) between the computational outcomes and the experimental assays collected from the bibliography [41]. Likewise, for conserved epitopes between SARS-CoV-2 and the new Khosta-2 virus, the correlations between the immunoinformatic predictions and wet lab assays analyzing 12 HLA class I alleles and 10 HLA class II alleles were also very high, reaching 90% and 93%, respectively (Figure 6) [42]. Overall, the correlation between the bioinformatics results and the experimentally detected epitopes in these two studies was 88% for HLA class I and 94% for HLA class II (Figure 6) [41,42]. It should be noted that this low percentage of predicted epitopes in our studies, which was not confirmed experimentally, may not necessarily have been an error in the bioinformatics prediction, because these epitopes may not have been tested and may be identified in future studies as being recognized by T lymphocytes. Thus, the high correlation found in our studies may even increase over time. Despite the good correlation between the prediction and experimental identification of T-cell epitopes demonstrated in these immunoproteomic studies, further refinement of bioinformatics algorithms with new experimental data is needed to ensure that predictions are as close as possible to immunological reality and can be used with the utmost confidence when dealing with new pandemics.

## 11. Contribution to the Control of Future Zoonotic Pandemics Through Immunoinformatics Tools: An Example

The analyses described above highlight the speed and low cost associated with immunoinformatic analyses. They represent a very flexible tool that can be used to explore the potential cross-protection of currently licensed vaccines, those in ongoing clinical trials, or even those in preclinical development. This strategy can even be used to prepare for possible pandemic zoonoses associated with viruses that are or have been pathogenic to humans.

A possible example of this preventive strategy is based on MERS-CoV, which is another betacoronavirus (like the previous sarbecoviruses) but belongs to the subgenus Merbecovirus. Since its emergence in Saudi Arabia in the spring of 2012, there have been several outbreaks limited to different countries in the Arabian Peninsula and South Korea, resulting in 943 deaths with a case fatality rate of 36% (https://www.emro.who.int/health-topics/mers-cov/mers-outbreaks.html, accessed on 1 November 2024). Due to the large evolutionary divergence between November the Merbecovirus and the Sarbecovirus subgenera, there are no conserved epitopes between MERS-CoV and SARS-CoV-1 or SARS-CoV-2 for any HLA class I or II allele [41]. Thus, neither currently licensed SARS-CoV-2 vaccines nor SARS-CoV-1 vaccines in Phase I clinical trials would be able to provide cross-protection against outbreaks generated by new MERS-CoV variants. In addition, some close relatives of MERS-CoV have been identified in bats in South Africa and in various pig populations in different countries around the world; like SARS-CoV-2, these variants use the angiotensin-converting enzyme 2 receptor to productively infect human cells [68]. Therefore, it is not inconceivable that new zoonotic diseases caused by merbecoviruses will emerge in humans as a consequence of mutational and recombinant events. A specific vaccine against MERS-CoV containing the spike protein encoded by a recombinant modified Ankara vaccinia virus is under development. In Phase I clinical trials, this vaccine construct demonstrated safety and tolerability by eliciting potent humoral and cell-specific immune responses [69]. Thus, immunoinformatics analyses such as those performed in our laboratory may be able to identify the T-lymphocyte epitopes conserved between the MERS-CoV vaccine and new potentially zoonotic merbecorivuses in humans. This could provide clues about whether this vaccine could be a first line of defense against these new viruses until a specific vaccine against them is developed.

This type of immunoinformatics strategy is currently being developed against several pathogens such as *Brucella* [70], and *S. aureus* [71] bacteria, and viruses such as chikungunya virus [72], influenza [73], hepatitis virus [74], and Rift Valley fever virus [75], and even for design of cancer immunotherapy [76].

## 12. Conclusions and Future Perspectives

Typically, studies experimentally analyzing cellular immune responses to a given pathogen and/or the efficacy of a test vaccine have used cohorts of a few hundred individuals, usually geographically localized and therefore representing only a very small fraction of the incredible polymorphism of the HLA gene complex. However, bioinformatics tools focusing on nearly 700 of the most common HLA alleles in the human population worldwide, such as those used in our laboratory to explore SARS-CoV-2 and other sarbecoviruses, can be very useful tools to obtain a global view of cellular immune responses and draw relevant conclusions that may even serve to reposition vaccines already on the market or contribute to clinical trials in the face of new pandemic challenges. In addition, these tools can help identify potential gaps in vaccine coverage due to HLA diversity, ensuring more complete protection in different populations. The integration of these bioinformatics approaches with experimental data may improve our understanding of immune response mechanisms and lead to more effective vaccine designs.
